# Risk Factors for Developing Critical Illness Polyneuropathy at a Referral Medical Center in Lebanon: A Prospective Cohort Study

**DOI:** 10.7759/cureus.95737

**Published:** 2025-10-30

**Authors:** Hassan Doumiati, Fatima Rawas, Nisrin Jaafar, Ali Ezzedine, Bahaa Bou Dargham, Ragheb Ismail, Ziad Koleilat, Raja Sawaya

**Affiliations:** 1 Neurology, American University of Beirut Medical Center, Beirut, LBN; 2 Neurology, University of Missouri, Columbia, USA; 3 Anesthesiology, Beirut Arab University, Beirut, LBN; 4 Anesthesiology, Hammoud Hospital University Medical Center, Beirut, LBN; 5 Internal Medicine, American University of Beirut Medical Center, Beirut, LBN

**Keywords:** critical illness polyneuropathy, electromyography (emg), intensive care unit, nerve conduction studies, thyroid dysfunction

## Abstract

Background

Critical illness polyneuropathy (CIP) is a common neuromuscular complication in critically ill patients and is associated with increased morbidity, prolonged mechanical ventilation, and delayed recovery. This study aims to identify clinical factors associated with the development or progression of CIP in critically ill patients admitted to a tertiary care center in Lebanon.

Methodology

This prospective cohort study included 70 adult patients (≥18 years) admitted to the intensive care unit (ICU) at the American University of Beirut Medical Center. Baseline demographics, medical history, and clinical data were collected upon admission. Nerve conduction studies (NCS) were conducted on day zero and repeated on day seven of ICU stay. Hospital course details, new medications, and complications were recorded throughout the observation period. Categorical variables were analyzed using the chi-squared test, while continuous variables were compared using the Mann-Whitney U test. A p-value <0.05 was considered statistically significant.

Results

Of the 70 patients enrolled, 46 were excluded due to inability to complete the follow-up NCS, primarily because of ICU stays shorter than seven days. The final analysis included 24 patients, among whom nine (37.5%) developed new or progressive CIP by day seven. Among all clinical variables analyzed, only thyroid dysfunction showed a statistically significant association with CIP development (p = 0.026). Notably, 32% of patients with CIP had underlying hypothyroidism or hyperthyroidism. Other commonly suspected factors, including corticosteroid use, beta-blocker therapy, and sepsis, were not statistically significant in this cohort.

Conclusions

Thyroid dysfunction was significantly associated with the development of CIP in critically ill patients in this study. These findings suggest that thyroid status may play a previously underrecognized role in the pathogenesis of CIP and highlight the potential value of routine thyroid screening in ICU patients. Further research involving larger, multicenter cohorts is warranted to validate these results and investigate the mechanisms linking thyroid dysfunction to neuromuscular complications in critical illness.

## Introduction

Critical illness polyneuropathy (CIP) is a length-dependent sensorimotor axonal polyneuropathy commonly observed in intensive care unit (ICU) patients. It is typically characterized by reduced compound muscle action potential (CMAP) and sensory nerve action potential (SNAP) amplitudes, with normal or mildly decreased conduction velocities. The prognosis of CIP tends to be worse than that of critical illness myopathy, with 45% to 75% of patients experiencing persistent weakness 6-12 months after ICU discharge. The pathophysiology of CIP is multifactorial, involving membrane depolarization, sodium channel dysfunction, hypoperfusion, and elevated extracellular potassium levels. Additionally, small nerve fiber involvement contributes to neuropathic pain. Electrophysiological findings such as axonal degeneration, reduced nerve response amplitudes, and denervation markers support the diagnosis [1].

CIP frequently occurs in patients with sepsis, acute respiratory distress syndrome (ARDS), systemic inflammatory response syndrome (SIRS), or multiorgan failure, and typically presents as generalized limb and respiratory muscle weakness, complicating ventilator weaning. Established risk factors include prolonged ICU stays, hyperglycemia, infections, and the administration of neuromuscular blockers or corticosteroids. Differentiating CIP from similar neuromuscular conditions, such as Guillain-Barré syndrome, is critical, as management strategies differ significantly [2].

Although CIP can be detected within 72 hours of ICU admission, it more commonly develops during the second week of the ICU stay. Diagnosis is based on electromyography, which reveals fibrillation potentials and alterations in motor unit morphology. Muscle and nerve biopsies may demonstrate signs of denervation, reinnervation, and widespread axonal degeneration [3].

Management of CIP is primarily supportive, focusing on risk factor mitigation, nutritional support, and physical rehabilitation. While no targeted pharmacologic therapy is currently available, intensive insulin therapy has been associated with improved outcomes, including reduced duration of mechanical ventilation and ICU length of stay. Early mobilization contributes to muscle preservation and functional recovery, although it does not impact mortality. Enteral nutrition is preferred over parenteral feeding, and supplementation with amino acids and omega-3 fatty acids shows potential in reducing inflammation and promoting muscle protein synthesis. Continued research is necessary to develop more effective treatments and improve long-term outcomes for patients with CIP [4].

Recognizing and diagnosing CIP is essential due to its clinical presentation of generalized areflexic weakness, which can mimic acute inflammatory demyelinating polyneuropathy, and its strong association with increased ICU morbidity and mortality. Early identification allows for timely intervention and may enhance recovery. The objective of our study is twofold: first, to prospectively identify potential risk factors associated with the development or progression of CIP; and, second, to assess the role of baseline characteristics, including age, sex, medical history, and primary admission diagnosis, along with ICU-related factors, in influencing CIP onset.

## Materials and methods

This prospective cohort study aimed to evaluate the development of CIP in critically ill patients. Eligible participants were adults aged 18 years or older admitted to critical care units at the American University of Beirut Medical Center, including the Intensive Care Unit, Coronary Care Unit, and Respiratory Care and Pulmonary Unit, within 24 hours of hospital presentation. Patients were excluded if they experienced cardiopulmonary arrest at presentation, were transferred from other hospital units, had been bedridden for more than 24 hours before ICU admission, or were admitted from nursing homes or daycare centers. Individuals with prior admissions to critical care units were also excluded.

Clinical data were collected at baseline and included demographic information and relevant medical history. All patients underwent nerve conduction studies (NCS) involving the peroneal, tibial, sural, median, and ulnar nerves. For each motor nerve, CMAP was recorded; for each sensory nerve, SNAP was assessed. Key electrophysiological parameters included amplitude, distal latency, area under the curve, conduction velocity, and F-wave latency for the tibial nerves.

Follow-up data were collected throughout the ICU stay and included hospital course details, medications administered, and complications. NCS assessments were repeated on day seven of ICU admission. The primary outcome was the development of CIP, defined by a reduction in CMAP and SNAP amplitudes over the observation period.

Statistical analyses were performed using the chi-squared test of independence for categorical variables and the Mann-Whitney U test for continuous variables. Informed consent was obtained from all conscious patients. For intubated or unconscious patients, consent was obtained from a family member. No harm or adverse events were reported in association with the study procedures. All statistical analyses were conducted using SPSS software, version 24 (IBM Corp., Armonk, NY, USA).

## Results

A total of 70 patients were included in the study and underwent NCS on day zero. Of these, 45 patients were excluded due to the absence of follow-up NCS on day seven, leaving 25 patients with complete data for analysis. Among the 25 patients who underwent NCS on day seven, 16 (64%) did not develop CIP, while nine (36%) were diagnosed with CIP. The analysis included various patient characteristics, such as demographics, admission-related factors, comorbidities, prior treatments, and ICU-related factors. A detailed breakdown of these characteristics is provided in Table 1.

**Table 1:** Patient characteristics.

Category	Characteristic	Details
Demographics	Age	-
	Gender	-
	Days of intensive care unit stay	-
Admission factors	Cause of admission	-
	Smoking status and history	-
	Alcohol intake	-
	Recent surgery	Type of surgery
Comorbidities	Diabetes mellitus	Years since diagnosis
	Chronic kidney disease	Years since diagnosis
	History of neuropathy	Years since diagnosis
	History of myopathy	Years since diagnosis
	History of neuromuscular disorders	Years since diagnosis
	Asthma	Years since diagnosis
	Chronic obstructive pulmonary disease	Years since diagnosis
	Thyroid dysfunction	-
	Hepatitis C virus infection	Years since diagnosis
	HIV infection	Years since diagnosis
	History of syphilis	Years since diagnosis
	Malignancy	Type and years since diagnosis
	History of central nervous system disease	-
Prior treatments	History of chemotherapy	Type
	History of radiation therapy	-
	Medication intake before admission	Including steroid and statin use
Intensive care unit-related factors	Duration of mechanical ventilation	Including mechanical ventilation within 24 hours of admission
	Systemic steroid use on admission	-
	Presence of infection on admission	Type and location

Descriptive statistics were used to summarize the demographic, clinical, and laboratory characteristics of the patients. Fisher’s exact test was employed for all categorical variables due to the small sample size, ensuring accurate estimation of significance. For continuous variables, the Mann-Whitney U test was used, as the data were not normally distributed. To account for multiple comparisons and reduce the risk of Type I error, the significance threshold (α = 0.05) was adjusted using the Bonferroni correction based on the number of variables tested within each group of analysis (Table 2).

**Table 2:** Adjusted alpha values displayed for each test.

Variable type	Number of tests	Adjusted alpha (Bonferroni)
Continuous (Mann-Whitney test)	23	0.00217
Categorical (Fisher’s exact test)	13	0.00385

A total of 25 patients were included in the study. The cohort was predominantly male 19 (76%), with a median age of 72 years (interquartile range (IQR) = 58.5-79). The most common causes of ICU admission were septic shock (5, 20%), respiratory failure (4, 16%), and dyspnea (3, 12%). Other presenting diagnoses included decreased level of consciousness (2, 8%), hemorrhagic shock (2, 8%), and single cases (1, 4% each) of cardiogenic shock, enteroviral encephalitis, headache, middle cerebral artery stroke, obstructed tracheostomy, severe hyponatremia, status epilepticus, and tumor lysis syndrome. None of the patients had a history of neuromuscular disease, myopathy, neuropathy, or asthma.

Categorical variables

There were no statistically significant associations between the development of critical illness neuropathy (CIN) and categorical variables, including gender (p = 1.0), smoking status (p = 0.864), alcohol intake (p = 1.0), recent surgery (p = 0.673), diabetes (p = 0.355), chronic kidney disease (p = 0.097), or the presence of coronary artery disease (p = 1.0). Likewise, the use of medications such as aminoglycosides (p = 1.0), colistin (p = 0.312), clindamycin (p = 0.36), vasopressors (p = 0.667), inotropes (p = 1.0), systemic steroids (p = 1.0), sedatives (p = 1.0), and analgesics (p = 0.26) was not significantly associated with CIN status (Table 3). The only categorical variable that showed a statistically significant association was thyroid dysfunction (p = 0.026), where 32% of patients had either hypo or hyperthyroidism.

**Table 3:** Categorical Variables listed with their corresponding p-values in relation to whether patients developed or had worsening CIN. Fisher’s exact test was employed for all categorical variables due to the small sample size, ensuring accurate estimation of significance. CIN = critical illness neuropathy; CKD = chronic kidney disease; PVD = peripheral vascular disease; CAD = coronary artery disease; COPD = chronic obstructive pulmonary disease; HCV = hepatitis C virus; HIV = human immunodeficiency virus; CNS = central nervous system; MV = mechanical ventilation

Variable	Category	Count (%)	P-value (Fisher’s exact test)
Gender	Male	19 (76%)	1.0
	Female	6 (24%)	
Smoking	Non-smoker	9 (36%)	0.864
	Current smoker	11 (44%)	
	Ex-smoker	5 (20%)	
Alcohol intake (n = 23)	Non drinker	20 (87%)	1.0
	Social drinker	2 (8.7%)	
	Heavy drinker	1 (4.3%)	
Recent surgery	Yes	7 (28%)	0.673
	No	18 (72%)	
History of diabetes	Yes	7 (28%)	0.355
	No	18 (72%)	
CKD	Yes	12 (48%)	0.097
	No	13 (52%)	
PVD	Yes	0 (0%)	-
	No	25 (100%)	
CAD (n = 24)	Yes	4 (16.7%)	1.0
	No	20 (83.3%)	
COPD	Yes	5 (20%)	0.621
	No	20 (80%)	
Thyroid dysfunction	None	17 (68%)	0.026*
	Hypothyroidism	6 (24%)	
	Hyperthyroidism	2 (8%)	
HCV	Yes	1 (4%)	1.0
	No	24 (96%)	
HIV	Yes	1 (4%)	1.0
	No	24 (96%)	
History of malignancy	Yes	10 (40%)	0.397
	No	15 (60%)	
CNS disease	Yes	5 (20%)	1.0
	No	20 (80%)	
MV in 24 hours	Yes	15 (60%)	1.0
	No	10 (40%)	
Clindamycin	Yes	0 (0%)	0.36
	No	0 (0%)	
Aminoglycosides	Yes	12 (48%)	1.0
	No	13 (52%)	
Colistin	Yes	5 (20%)	0.312
	No	20 (80%)	
Infection on admission	Yes	17 (68%)	0.182
	No	8 (32%)	
Central line	Yes	16 (64%)	0.671
	No	9 (36%)	
Amiodarone	Yes	3 (12%)	1.0
	No	22 (88%)	
Calcium channel blocker	Yes	2 (8%)	1.0
	No	23 (92%)	
Systemic steroids	Yes	8 (32%)	1.0
	No	17 (68%)	
Sedatives	Yes	6 (24%)	1.0
	No	19 (76%)	
Analgesics	Yes	7 (28%)	0.26
	No	18 (72%)	
Colchicine	Yes	0 (0%)	-
	No	25 (100%)	
Vasopressors	Yes	16 (66.7%)	0.667
	No	8 (33.3%)	
Inotropes	Yes	1 (4%)	1.0
	No	24 (96%)	
Beta-blocker	Yes	13 (52%)	0.226
	No	12 (48%)	

Continuous variables

Among continuous variables, serum creatinine (Cr) was significantly higher in patients without CIN (median = 1.50 mg/dL, IQR = 0.90-2.50, p = 0.024). Other renal, hepatic, inflammatory, and metabolic markers, including phosphorus (p = 0.076), magnesium (p = 0.098), calcium (p = 0.364), alanine transaminase (p = 0.325), aspartate transaminase (p = 0.510), albumin (p = 0.716), C-reactive protein (p = 0.336), and lactic acid (p = 0.953), did not differ significantly between groups (Table 4). There were no significant differences in total ICU stay (p = 0.967), duration of mechanical ventilation (p = 0.103), or systemic steroid dose (p = 0.592) between groups.

**Table 4:** Continuous variables with their medians in relation to whether patients developed CIN or had CIN worsening. For continuous variables, the Mann-Whitney U test was used, as the data were not normally distributed. CIN = critical illness neuropathy; ICU = intensive care unit; MV = mechanical ventilation; AST = aspartate aminotransferase; ALT = alanine aminotransferase; ALP = alkaline phosphatase; GGT = gamma-glutamyl transferase; CRP = C-reactive protein; INR = international normalized ratio; ESR = erythrocyte sedimentation rate; CPK = creatine phosphokinase; TSH = thyroid-stimulating hormone

Variable	Median (IQR)	P-value (Mann-Whitney test)	Mann-Whitney U value
Age (years)	72.00 (58.50–79.00)	0.513	60
Total ICU stay	57.00 (26.00–149.00)	0.967	71
Duration of MV (days)	11.00 (0.00–27.00)	0.103	31.5
Steroid equivalent dose as Prednisone (mg/24 hour)	75.00 (75.00–75.00)	0.592	37
Potassium	4.30 (3.90–5.20)	0.879	69
Calcium	8.50 (7.90–9.10)	0.364	55.5
Phosphorus	3.70 (2.75–5.00)	0.076	40.5
Magnesium	2.00 (1.75–2.45)	0.098	42.5
Creatinine	1.50 (0.90–2.50)	0.024*	32.5
ALT	31.50 (13.75–58.75)	0.325	50.5
AST	29.00 (22.50–88.50)	0.51	56
ALP	105.00 (82.00–163.00)	0.591	51
GGT	43.00 (25.00–81.75)	0.163	27.5
Total bilirubin	0.55 (0.375–0.975)	0.408	43.5
Albumin	27.00 (20.00–36.00)	0.716	54
CRP	58.20 (28.00–189.30)	0.336	47
Lactic acid	2.19 (1.60–3.62)	0.953	66
INR	1.20 (1.10–1.70)	0.47	59
ESR	35.00 (23.00–46.00)	0.561	10.5
CPK	56.00 (45.50–265.00)	0.245	20.5
TSH level	1.415 (0.505–2.410)	0.341	36.5
Vitamin B12 (pg/mL)	498.00 (431.00–1,490.00)	0.931	14
25-hydroxyvitamin D	10.90 (7.20–27.40)	0.336	19

## Discussion

In our cohort of critically ill patients with CIN, thyroid dysfunction was the only categorical variable significantly associated with the development or progression of the condition. This finding suggests a potentially unique and important role of thyroid function in CIN pathophysiology. Notably, other demographic and clinical factors, including gender, common comorbidities such as diabetes or chronic kidney disease, and frequently used ICU medications such as systemic steroids, vasopressors, or sedatives, showed no significant association with CIN. Similarly, ICU-related factors such as length of stay and duration of mechanical ventilation did not differ significantly between groups. While serum creatinine was higher in patients without CIN, this inverse relationship lacks clear clinical significance and may be influenced by sample size limitations or confounding variables. Overall, these results emphasize thyroid dysfunction as a key factor in CIN development, warranting further exploration in larger, prospective cohorts to validate these findings and elucidate underlying mechanisms.

The pathophysiology of CIP primarily involves axonal degeneration of motor and sensory fibers, triggered by several interrelated mechanisms. A key factor is hypoperfusion, which leads to hypoxia and decreased nutrient delivery to peripheral nerves, resulting in ischemic and metabolic injury. Typically, the onset of nerve conduction abnormalities begins one to two weeks after the development of circulatory compromise, such as in multiorgan failure or systemic inflammation. In addition, electrolyte imbalances and renal dysfunction in critically ill patients may lead to elevated extracellular potassium, which further impairs nerve excitability. Advanced electrophysiologic studies have shown that in CIP, motor axons exhibit membrane depolarization and inactivation of voltage-gated sodium channels, indicating an acquired sodium channelopathy that disrupts normal action potential propagation. Furthermore, small fiber involvement, demonstrated by intraepidermal nerve fiber loss and impaired sweat gland innervation, suggests that both large and small fibers are affected [1].

Additional mechanisms include microvascular dysfunction at the level of the vasa nervorum, where increased permeability, potentially driven by elevated E-selectin expression, leads to immune cell infiltration, release of inflammatory cytokines such as tumor necrosis factor-alpha and interleukin-1, and the development of endoneurial edema. This edema contributes to local hypoxia, disrupting mitochondrial function, impairing ATP production, and triggering the formation of reactive oxygen species (ROS). The accumulation of ROS causes oxidative stress and structural mitochondrial damage, further amplifying axonal injury [4]. Additionally, hyperglycemia in critical illness exacerbates nerve damage by promoting intracellular glucose overload and enhanced ROS production, particularly superoxide and peroxynitrite, while also impairing ROS scavenging mechanisms. These effects together contribute to bioenergetic failure, ion channel dysfunction, and neuronal apoptosis, culminating in the characteristic diffuse axonal degeneration seen in CIP [4].

The role of thyroid hormones in nerve health is well supported by existing literature. In patients with newly diagnosed hypothyroidism or hyperthyroidism, neuromuscular symptoms are common. A prospective study found that 42% of hypothyroid patients had electrophysiological evidence of sensorimotor axonal neuropathy. Treatment leading to restoration of euthyroid status reduced neuropathic findings [5]. This is relevant because it provides a model in which thyroid hormone status directly influences peripheral nerve integrity.

Previous literature has also demonstrated similar associations, supporting the link between thyroid dysfunction and critical illness neuropathy. The hypothalamic-pituitary-thyroid axis tightly regulates thyroid hormone levels via a feedback loop, but critical illness disrupts this balance, leading to the non-thyroidal illness syndrome (NTIS). NTIS is characterized by low circulating T3 and T4 despite normal or slightly decreased thyroid-stimulating hormone, reflecting an adaptive response to systemic stress and inflammation. Given the essential roles of thyroid hormones in cellular metabolism, nerve function, and tissue repair, dysregulation of this axis can plausibly increase peripheral nerve vulnerability during critical illness. This biological framework provides a mechanistic explanation supporting our cohort’s observation linking thyroid dysfunction with CIN. The metabolic and inflammatory disturbances associated with NTIS may impair nerve regeneration and worsen neurodegeneration, positioning thyroid dysfunction as a modifiable contributor to CIN pathogenesis [6-8].

The study reported by Grigorescu and Fodor further corroborated these findings by describing neuroendocrine alterations in critical illness, particularly suppression of the thyroid axis, that likely contribute to CIN development. According to Grigorescu and Fodor, the thyroid hormone imbalance characteristic of NTIS, combined with the triad of critically ill factors, inflammation, coagulopathy, and hormonal disruption, exacerbates cellular stress and compromises peripheral nerve function or repair. This integrative perspective from the literature strengthens the biological plausibility of thyroid dysfunction as a significant player in the complex pathophysiology of CIN, aligning closely with our cohort’s results and suggesting new avenues for therapeutic intervention [9].

Shabana et al. highlighted a significant association between lower free triiodothyronine (FT3) levels and the development of intensive care unit-acquired weakness (ICUAW). NTIS represents a dysregulated hormonal response to critical illness. This adaptive response may become maladaptive, contributing to impaired muscle metabolism, catabolism, and weakness. While Shabana et al. focused on ICUAW, our cohort study found a similar association between thyroid dysfunction, particularly NTIS, and the development of CIN. This parallel supports the hypothesis that thyroid hormone disruption plays a broader role in the pathophysiology of neuromuscular complications in critically ill patients, reinforcing the need for further research into thyroid status as a modifiable risk factor [10].

Persisting neuroendocrine abnormalities are also linked to long-term physical impairment after critical illness. For instance, a study of ICU survivors found that even five years after ICU discharge, disturbances in the thyroid axis remained in many patients, particularly elevated reverse T3 and a low T3/rT3 ratio; these were associated with poorer physical capacity (e.g., reduced handgrip strength) [11]. These findings suggest that the impact of thyroid dysfunction may extend beyond the acute ICU phase and influence long-term neuromuscular recovery.

While previous literature has identified multiple risk factors and etiologies for CIP, our cohort did not demonstrate these associations. The development of CIP is multifactorial and primarily linked to systemic consequences of critical illness. Frequently cited risk factors include sepsis, systemic inflammatory response syndrome, multiorgan dysfunction syndrome, multiorgan failure, female sex, corticosteroid use, severe asthma, electrolyte disturbances, malnutrition, immobility, and hyperglycemia. Sepsis and multiorgan dysfunction syndrome have long been considered central to CIP, with reported incidence rates as high as 70% and up to 100% in multiorgan failure cases; however, some large studies have questioned a direct causal relationship [12]. Hyperglycemia remains a consistent risk factor, with intensive insulin therapy demonstrating a reduction in CIP incidence, likely due to its neurotoxic effects and mitochondrial dysfunction [12]. Female gender, longer duration of mechanical ventilation, use of vasopressor, renal replacement therapy, cardiothoracic surgery, glucocorticoids, and neuromuscular blockers have all been reported but are inconsistent across studies [3,12]. Overall, systemic inflammation, microvascular compromise, and metabolic disturbances underpin CIP pathogenesis, but specific risk factors may vary depending on patient populations.

This study benefits from a prospective design, allowing systematic and timely data collection on critically ill patients from the point of ICU admission. The inclusion of NCS performed at baseline and repeated after seven days provides objective and quantitative measures for diagnosing and tracking the development of CIP. The use of standardized electrophysiological parameters across multiple motor and sensory nerves strengthens the reliability of CIP detection. Furthermore, the comprehensive collection of clinical, demographic, and medication data allows for a robust analysis of potential risk factors associated with CIP. The strict inclusion and exclusion criteria help minimize confounding by prior neurological or critical care exposures, thus improving the internal validity of the findings. Additionally, the study’s focus on thyroid dysfunction aligns with emerging literature, providing biological plausibility to the observed association and highlighting a potentially modifiable factor in CIP pathogenesis.

Despite these strengths, the study has several limitations. The relatively small sample size may limit the statistical power to detect associations with less prominent risk factors and restrict the generalizability of the findings. The single-center setting may also limit external validity, as patient populations and ICU practices can vary across institutions. Although NCS are the gold standard for CIP diagnosis, they can be technically challenging in critically ill patients and may be influenced by factors such as edema or limb positioning, potentially affecting measurement accuracy. Additionally, the follow-up period of seven days may not capture the full course or the late onset of CIP in some patients. Finally, while the study carefully excluded patients with prior neuromuscular disease or prolonged immobilization, residual confounding from unmeasured variables cannot be entirely ruled out.

Further research with larger, multicenter cohorts is needed to validate and expand upon our findings, allowing for greater generalizability across diverse ICU populations. Incorporating longer follow-up periods and additional biomarkers could provide deeper insights into the temporal dynamics and mechanistic pathways of CIP.

## Conclusions

Thyroid dysfunction appears to be a meaningful risk factor for the development of CIP in patients in ICU settings. In contrast, demographic factors, comorbidities, and commonly used medications did not show strong predictive value in our cohort. Although our study is limited by its modest sample size and single-center design, the findings suggest that neuroendocrine disturbances, especially those involving thyroid regulation, may contribute to nerve vulnerability during critical illness. Further multicenter studies with larger and more diverse populations are needed to validate this association and investigate whether modulating thyroid function might mitigate neurologic complications in critically ill patients. Overall, attention to thyroid status may inform future strategies to preserve neuromuscular health in this vulnerable population.
